# Examining the spatial risk environment tied to the opioid crisis through a unique public health, EMS, and academic research collaborative: Lowell, Massachusetts, 2008–2018

**DOI:** 10.1016/j.pmedr.2021.101591

**Published:** 2021-10-06

**Authors:** Thomas J. Stopka, Erin Jacque, Jon Kelley, Lainnie Emond, Kerran Vigroux, Wilson R. Palacios

**Affiliations:** a Dept. of Public Health and Community Medicine, Tufts University School of Medicine, United States; b Trinity Emergency Medical Services, United States; c Lowell Department of Health, United States; d School of Criminology & Justice Studies, University of Massachusetts, Lowell, United States

**Keywords:** Opioids, Overdose, Syringe discard, Lowell, Massachusetts

## Abstract

Between 2015 and 2018, Lowell Massachusetts experienced outbreaks in opioid overdoses, HIV, and hepatitis C virus infections (HCV) among people who inject drugs. Through an innovative collaboration between emergency medical services (EMS), public health, and academic partners, we assessed the geographic distribution of opioid-related risks to inform intervention efforts. We analyzed data from three unique data sources for publicly discarded syringes, opioid-related incidents (ORIs), and fatal opioid overdoses in Lowell between 2008 and 2018. We assessed the risk environment over time using a geographic information system to identify and characterize hotspots and noted parallel trends within the syringe discard and ORI data. We identified two notable increases in ORIs per day: the first occurring between 2008 and 2010 (from 0.3 to 0.5), and the second between 2011 and 2014 (from 0.9 to 1.3), following the introduction of fentanyl within local drug markets. We also identified seasonal patterns in the syringe discard, ORI, and overdose data. Through our spatial analyses, we identified significant clusters of discarded syringes, ORIs, and fatal overdoses (p < 0.05), and neighborhoods where high densities of these outcomes overlapped. We found that areas with the highest densities shifted over time, expanding beyond the epicenter of the Downtown neighborhood. Data sharing and analyses among EMS, public health, and academic partners can foster better assessments of local risk environments. Our work, along with new public health efforts in Lowell, led to a city-funded position to improve pick-up and proper disposal of publicly discarded syringes, and better targeted harm reduction services.

## 1 Introduction

The opioid crisis has been one of the greatest public health challenges during the past two decades. In Massachusetts, there was a five-fold increase in fatal overdoses between 2000 and 2018, from 375 to 2,005, respectively (MDPH, 2021), and the state ranked 7th in the country for opioid overdose death rates in 2017 (Rudd et al., 2016, MDPH, 2016). More than 65,000 nonfatal opioid overdoses occurred in Massachusetts between 2011 and 2015, with more than 20,000 in 2015 alone (An Assessment of Fatal and Non-Fatal Opioid Overdoses in Massachusetts, 2017). Infectious complications attributed to injection drug use have also increased across the state. Confirmed chronic hepatitis C virus (HCV) cases per year increased from 4449 in 2014 to 4742 in 2018, with notable increases in HCV counts and rates among 30–39 year-olds from 2014 (1730; 210.5 per 100,000) to 2018 (2043; 248.6 per 100,000) (MDPH, 2020). As of January 2019, 21,612 people were living with HIV in Massachusetts (2019 Massachusetts HIV/AIDS Epidemiologic Profile, 2020). Between 2015 and 2018, an HIV outbreak was reported, largely among people who inject drugs (PWID), in the cities of Lowell and Lawrence, in the Northeastern region of the state (Cranston et al., 2019, Alpren et al., 2020). Recent public health and clinical advisories have indicated that new HIV cases were also diagnosed in late 2018 and early 2019 in Boston, raising concerns of additional epidemics among PWID extending to other locations (Fryer, 2019).

Previous studies have assessed local syringe discard patterns in local communities. Investigators in Massachusetts and Connecticut found that neighborhoods with more stable economic status were associated with more safe syringe discard practices compared to less advantaged neighborhoods (Buchanan et al., 2003). Another study compared syringe discard patterns in San Francisco, which had longstanding full-time syringe services programs (SSPs), to those in Miami, where SSPs were not in place; findings indicated that PWID in Miami had 34 times increased odds of public syringe disposal compared to PWID in San Francisco (Tookes et al., 2012). Other recent studies have depicted the importance of using publicly available data to evaluate syringe discard patterns in local communities to better understand local opioid overdose epidemics (Bearnot et al., 2018), as well as seasonal variations in risk (Sadler and Furr-Holden, 2019), implications for syndemic risks, and triangulation of treatment services for injection-mediated comorbidities (Furr-Holden et al., 2016). While an increasing number of studies have begun to focus on analysis of emergency medical services (EMS) data (Garza and Dyer, 2016, Knowlton et al., 2013, Moore et al., 2017), there is a burgeoning need for collaborative efforts between local EMS, public health, and academic partners to respond to syringe discard challenges, and to use public syringe discard patterns, EMS, and vital statistics data to identify neighborhoods at highest risk for opioid overdose, HIV and HCV infections. Gaps in overdose, HIV, and HCV surveillance systems need to be filled using local data from unique sources to gain a better understanding of neighborhood-level risks.

Lowell is a post-industrial city of over 108,000 residents, experiencing decades-long challenges with structural poverty and economic distress (Bureau UC, 2021). The opioid crisis has been particularly devastating in Lowell where, in 2016, the fatal overdose rate was more than twice that of the Massachusetts statewide rate (63 per 100,000 population vs. 30.6, respectively) (MDPH, 2021, MDPH, 2019).

The goal of this study was to assess the spatial risk environment and compare trends in syringe discard, opioid-related illnesses, and fatal overdoses to identify hotspots for injection-mediated risks, and seasonal patterns, in Lowell. As part of a larger community collaboration initiated in December 2017, we highlight the benefits of collaborative efforts between local EMS leaders, public health officials, and academic researchers to link unique data sources, and monitor and respond to opioid-related incidents, with the ultimate goal of informing targeted and enhanced interventions, curbing local opioid overdose and HIV epidemics.

## 2 Methods

Data. We compiled data for four unique outcomes: (1) publicly discarded syringe reports, (2) opioid-related incidents (ORIs), (3) injury location of fatal overdose and (4) fatal opioid overdose death locations. The injury and fatal overdose location data were obtained from the same data source, as described below. We obtained population data from the City to calculate rates per 10,000.

*Syringe Discard Data.* We obtained data for public syringe discard reports for 2011 to 2018 from Trinity EMS, a 9-1-1 emergency and non-emergency ambulance and wheelchair van transportation service provider in the Merrimack Valley of Massachusetts and Southern New Hampshire. Public syringe discard data represent calls made to Trinity EMS to report a discarded syringe(s) at the address level. Calls typically result in the retrieval and safe disposal of one or more syringes.

*ORIs.* We obtained ORI data for 2008–2018. Suspected ORIs were defined as incidents in which EMS interacted with patients and opioid use was witnessed, reported, or suspected. Beginning in 2013, Confirmed ORIs (C-ORIs) were documented in Lowell. C-ORIs represented ORIs that were reviewed by Trinity EMS data specialists to verify that the ORI met the working definition. For each interaction a Trinity EMS crew member has with a patient, an electronic Patient Care Report (ePCR) is generated. The ePCR contains data points that are scanned for keywords by a computer program called FirstWatch. FirstWatch is programmed to scan for keywords including: heroin, opioid, opium, Narcan, naloxone, needle, syringe, overdose-heroin, and overdose-opioid. When FirstWatch identifies a keyword in an ePCR, an alert is automatically sent to authorized users, including designates at the Fire, Health, and Police Departments. FirstWatch tracks the number of ORIs during the past 72 h. When there are greater than 20 ORIs in a 72-hour period the “Lowell Surge Protocol” is enacted. This protocol prompts discussions with City Leadership, Police and Fire Chiefs, and EMS. Response actions include mass communications and warnings from the City, which can help trigger enhanced prevention (e.g., overdose education and naloxone distribution), follow-up (i.e., with recovery coaches) and treatment responses.

Trinity EMS plays a critical role in the tracking and dissemination of ORI data for Lowell. 100% of ORIs that are reported through 9–1-1 calls in Lowell are captured and analyzed by Trinity EMS. Trinity EMS has been the primary source of ORI data for Lowell since 2008, and shares data with community partners to track dozens of ORI data points.

*Injury Locations.* These locations identified the address where the decedent was found injured, due to an opioid overdose, prior to death. We obtained addresses of injury locations for opioid overdose decedents for 2015–2017 from the Massachusetts Registry of Vital Records and Statistics (RVRS).

*Fatal Overdoses.* We also obtained addresses of death locations for 2015–2017 from RVRS.

*Sociodemographic Measures.* We downloaded publicly available data from the U.S. Census Bureau’s American Community Survey (United States Census Bureau, 2011). We compiled 5-year estimates for total population, race, and poverty measures at the census tract level.

Descriptive Statistics. We ran frequencies on syringe discard, ORI, and fatal overdoses to assess measures of central tendencies and trends. We aggregated ORI counts and syringe discard reports on a monthly level, from January 2008 through December 2018. We also assessed ORIs and fatal overdoses by season.

Geocoding. We geocoded address-level data for syringe discard reports and opioid overdoses in Google My Maps and ArcMap. We obtained a geocoding match rate of 99.4% for syringe discard reports and 99% for fatal overdoses. For syringe discards, many locations were denoted at the nearest street intersections. Google My Maps provided a higher match rate for geocoded street intersections. ORI data were not geocoded and mapped at the sub-county-level to protect the confidentiality of the people who survived them.

GIS Mapping. We developed thematic maps for total population, percent non-white population, and percent poverty status by census tract.

Spatial analyses. We calculated annual Kernel density estimates (KDE), producing a total of fourteen “heat maps”. For our KDE analyses, we used square miles as the area unit, and a search radius of 402.336 m (or ¼ square mile), considered as a standard walking distance in urban areas (Milam et al., 2012, Furr-Holden et al., 2016). The City of Lowell boundary polygon was used as the processing extent (ArcGIS-Pro. How Kernel Density Works., 2020). Once density raster’s were created for fatal overdose death locations, public syringe discard reports, and injury address locations for 2015, 2016, and 2017, a composite raster was created using the raster calculator that combined densities for 9 raster datasets. A composite raster was also developed that excluded the death locations that occurred in hospitals. We also conducted optimized hotspot cluster analyses to identify significant clusters of discarded syringes (2011–2018), fatal overdose deaths (2015–2017) both with and without deaths that occurred in hospitals, and injury locations (2015–2017). Significant clusters are represented on the maps by 90%, 95%, and 99% confidence intervals. The optimized hotspot analysis tool generalizes and automates the manual 5-step geoprocessing approach used in previous studies (Stopka et al., 2014, Stopka et al., 2019). Through use of this tool, we aggregated incidents into weighted features and, using the distribution of the weighted features, identified an appropriate scale of analysis, and ultimately calculated the Getis-Ord Gi* statistic, adjusted for multiple testing and spatial dependence using the false discovery rate correction (ArcGIS-Pro, 2020). All final maps were created in ArcGIS 10.7.1 (Esri, Redlands, CA). Study procedures were approved by the Tufts University Health Sciences Institutional Review Board.

## 3 Results

Syringe discard rates per 10,000 population were highest for the Downtown (1884), Back Central (946), Lower Belvidere (523), Acre (398), and Centralville (356) neighborhoods. ORI rates were highest for the Downtown (2338), Back Central (918), Acre (521), Centralville (400), and Lower Belvidere neighborhoods (349). The ORI rate in the Downtown neighborhood was 2.5 times greater than that in the Back Central neighborhood. The syringe discard rate in the Downtown neighborhood was almost two times greater than that in the Back Central neighborhood (Table 1).

**Table 1:** Counts and rates per 10,000 for opioid-related incidents (ORI) (2008–2018), syringe discard reports (2011–2018), and fatal opioid overdoses (2015–2017) across Neighborhoods in Lowell, Massachusetts.

Neighborhoods	Pop.	ORI	ORI Rate per 10,000	Syringe Discard Reports	Syringe Discard Report Rate per 10,000	Average Annual Fatal OD Count	Average Annual Fatal OD Rate
Acre	12,903	672	521	514	398	7.7	5.9
Back Central	5667	520	918	536	946	6.3	11.2
Belvidere	9710	135	139	134	138	2.0	2.1
Centralville	15,237	610	400	543	356	7.7	5.0
Downtown	4543	1062	2338	856	1884	3.3	7.3
Highlands	18,312	301	164	213	116	6.3	3.5
Lower Belvidere	2354	108	459	123	523	7.0	29.7
Lower Highlands	11,878	414	349	264	222	4.3	3.6
Pawtucketville	15,020	273	182	165	110	15	10.0
Sacred Heart	7250	191	263	133	183	2.0	2.8
South Lowell	3645	64	176	47	129	1.3	3.7

Abbreviations: Pop. = Population; ORI = Opioid-related incidents; OD = overdose.

**Fig. 1:**
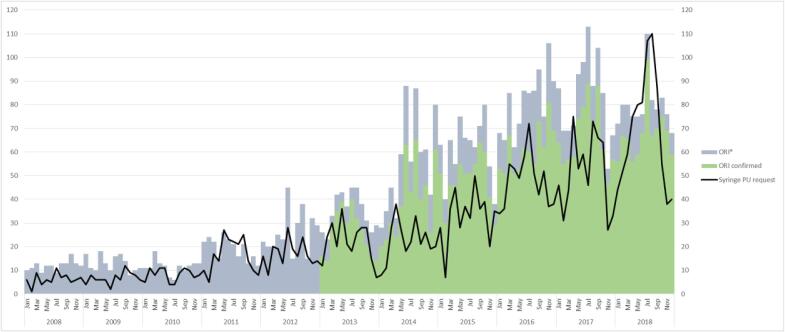
Opioid related incident (ORI) calls and public syringe pick-up (PU) requests received by Trinity EMS, Lowell, Massachusetts, 2008–2018.

**Fig. 2:**
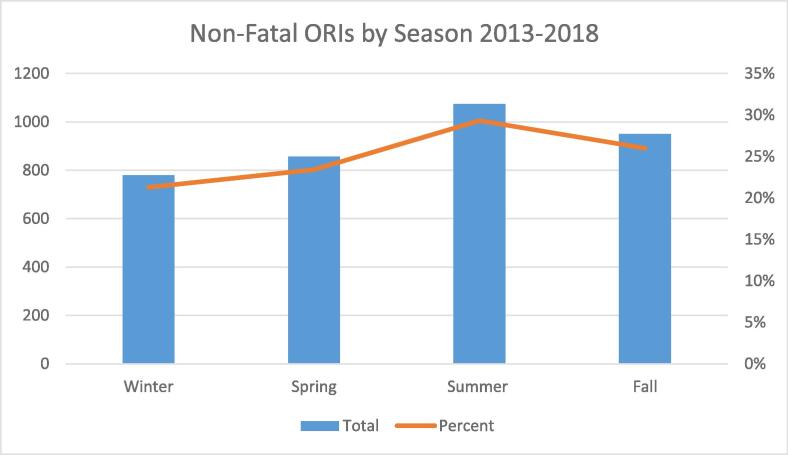
Number of opioid-related incident (ORI) calls and percentage of all 911 calls that were ORI calls by season, Lowell, Massachusetts, 2013–2018.

After 2008, both ORIs and discarded syringe pick-up requests increased steadily. During the first quarter of 2011 and the second quarter of 2014, steep increases were noted, both of which remained unabated through December of 2018. We identified annual temporal patterns in syringe pick-up reports, with peaks in the summer and valleys in the winter (Fig. 1). Similarly, we noted seasonal differences in ORIs and fatal overdoses, with elevated measures during the summer (Fig. 2).

## 4 Spatial analyses

### 4.1 Population, race and poverty

#### 4.1.1 Thematic maps

**Fig. 3:**
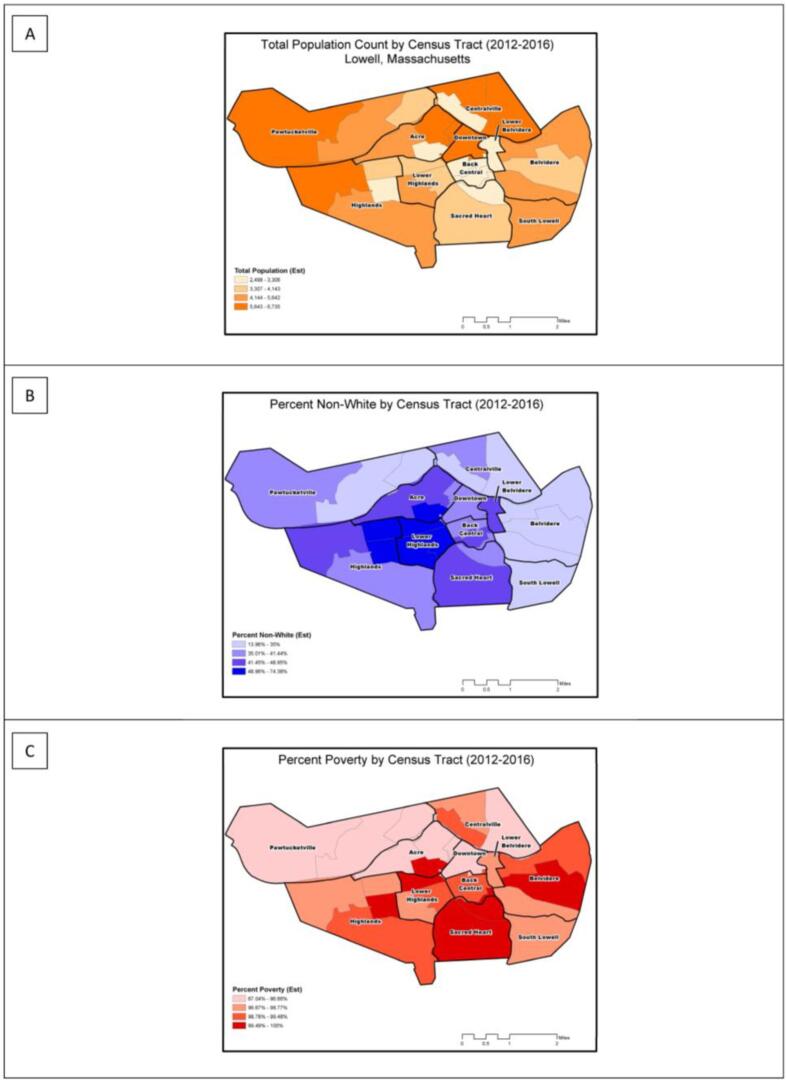
Sociodemographic characteristics of Lowell neighborhoods, 2012–2016: (A) Total population by census tract; (B) percent non-white population by census tract; (C) percent the population living in poverty. All maps are classified using quartiles.

We found that total population in Lowell was greatest in the census tracts in the western Pawtucketville, Highlands, northeast Acre, Downtown and Centralville neighborhoods (Fig. 3a). We found the non-white population (>50%) to be most concentrated within in the Lower Highlands, northeast Highlands, and southcentral Acre neighborhoods (Fig. 3b). Poverty in Lowell was widespread, but percentages were highest in the southern half of the city (Fig. 3c).

### 4.2 Public syringe discard reports

#### 4.2.1 Descriptive maps

Syringe discard reports were most concentrated in central Lowell, Downtown, Back Central, Lower Highlands, Acre, and Centralville neighborhoods. In some areas, the distribution of points appeared to follow a linear distribution along transportation corridors/roads (Appendix).

#### 4.2.2 KDEs (heat maps)

We calculated KDEs for each year from 2011 to 2018 (Appendix). The resulting heat map rasters from the 2015–2017 analyses were incorporated into multi-variable composite density maps.

#### 4.2.3 Hotspot clusters

**Fig. 4:**
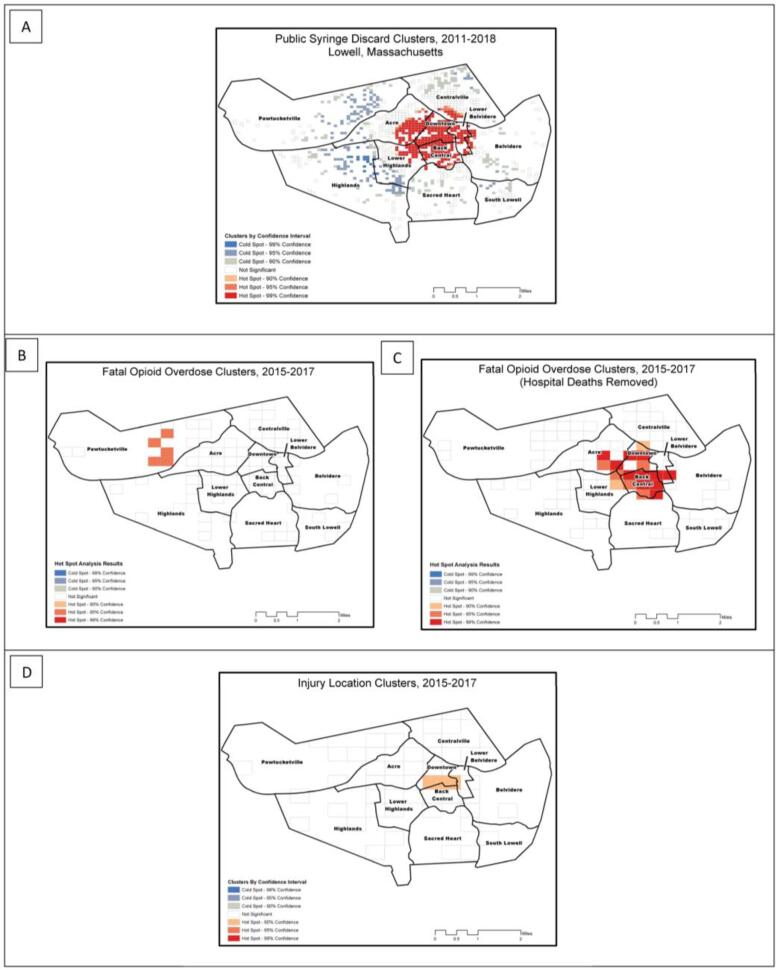
Optimized hotspot cluster analyses in Lowell Massachusetts, 2015–2017: (A) Public syringe discard clusters, 2011–2018; (B) fatal opioid overdose clusters, 2015–2017; (C) fatal opioid overdose clusters, 2015–2017 (hospital deaths removed from analysis); (D) decedent injury locations, 2015–2017.

We identified a hotspot for syringe discard for 2011–2018 located in the Downtown, Back Central, and Lower Belvidere neighborhoods (p < 0.05) (Fig. 4a).

Fatal Opioid Overdose

#### 4.2.4 Descriptive maps

Some fatal overdose locations shared the same street address. To more accurately display the number of fatal overdoses, we used graduated symbols to highlight the locations where multiple fatal overdoses were reported (Appendix).

#### 4.2.5 Heat maps

We created heat maps for 2015–2017 for all fatal overdose locations and fatal overdose locations with hospital points removed (Appendix). Both sets of resulting heat map rasters were incorporated into multivariable composite density maps.

#### 4.2.6 Hotspot clusters

Through our cluster analyses for fatal overdose locations from 2015 to 2017, we identified hotspots in the Pawtucketville neighborhood around the Lowell General Hospital (p < 0.05), extending to the Acre neighborhood (Fig. 4b). Through cluster analysis, after removing fatal overdoses that were recorded in hospitals, (Fig. 4c) we identified hotspots in the Back Central, Downtown, Acre, Lower Highlands, Sacred Heart, Belvidere, Lower Belvidere and Centralville neighborhoods (p < 0.05).

Injury Location

#### 4.2.7 Heat maps

We calculated KDEs for 2015–2017 (Appendix) for use in the multivariable composite density map.

#### 4.2.8 Hotspot clusters

Through cluster analysis of injury location (Fig. 4d), we identified a hotspot that straddled the Downtown, Back Central and Lower Belvidere neighborhoods.

### 4.3 Composite density results: public syringe discard, fatal overdose, and injury locations

#### 4.3.1 Composite KDEs (hospital death points included)

**Fig. 5:**
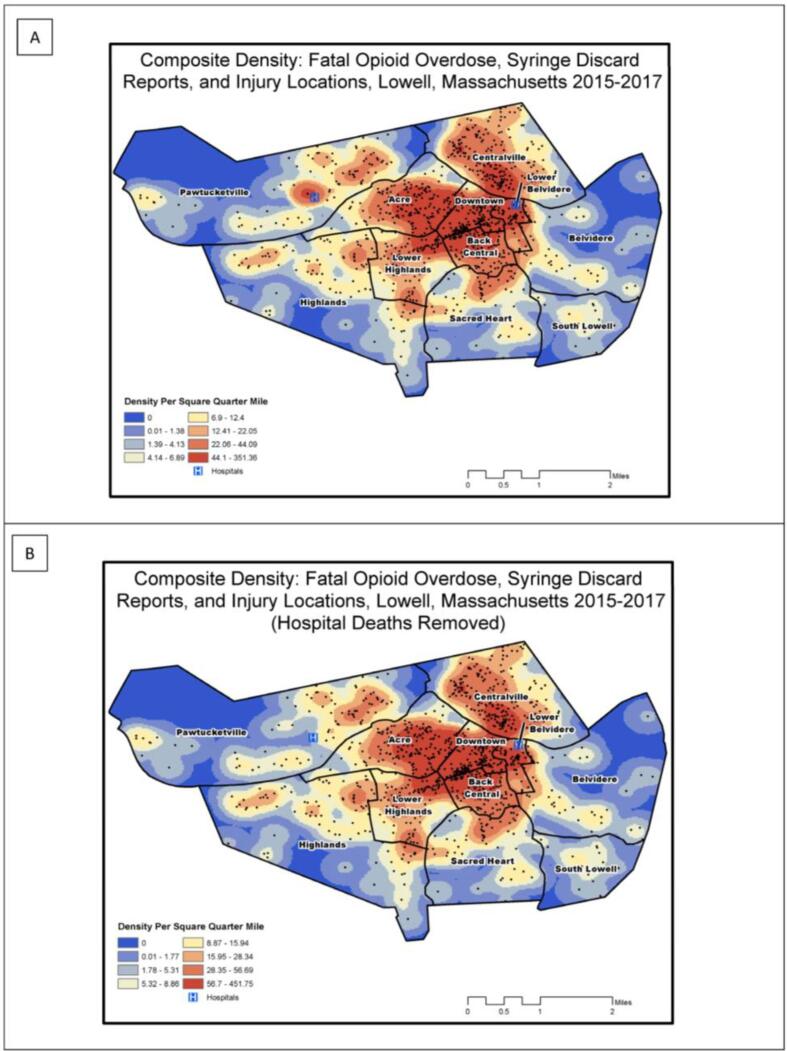
Density of opioid-related risks, Lowell, Massachusetts, 2015–2017: (A) Composite raster that sums the densities per square ¼ mile of fatal opioid overdose, public syringe discard, and decedent injury locations for 2015, 2016, 2017; (B) Composite raster that sums the same densities in Fig. 3A, without including hospital-recorded deaths. Maps are classified using Octiles.

Through composite density calculations (Fig. 5a), we identified the highest density areas in the Downtown, Lower Belvidere, Centralville, Lower Highlands, and Back Central neighborhoods. High density areas were also present, to a lesser extent, in the Highlands, Sacred Heart, and Belvidere neighborhoods. The Pawtucket neighborhood had a prominent high-density location depicting the deaths reported at Lowell General Hospital, as well as two other high-density areas to the east.

#### 4.3.2 Composite KDEs (hospital death points excluded)

Through composite density calculations without hospital deaths, we identified similar density distributions across most neighborhoods (Fig. 5b), however the prominent high-density location in Pawtucketville was no longer present.

## 5 Discussion

We used a unique mix of data to assess the opioid crisis in Lowell, Massachusetts between 2011 and 2018, when spikes occurred in fatal overdoses, ORIs, and new HIV and HCV infections among PWID, largely attributed to the entry of fentanyl in local drug markets (Cranston et al., 2019, Alpren et al., 2020).

Through spatial and trend analyses, we noted similar geographic distributions in syringe discards, ORIs, and fatal overdoses. Since these variables could be considered proxies for risk, we identified neighborhoods where public health interventions (e.g., enhanced harm reduction and drug treatment services) should be prioritized. In our trend analyses, we documented two notable increases; the first occurring during the first quarter of 2011 and the second during the second quarter of 2014. A significant tipping point in the opioid crisis was the introduction of fentanyl during 2013 (Somerville et al., 2017, Ciccarone et al., 2017), which increased incidence and prevalence of drug-related harms. Similar to results for Northeastern states from Sadler and Furr-Holding, we also found seasonal variation in overdose risk (Sadler and Furr-Holden, 2019), with increased risk during summer months.

Through our spatial analyses of EMS and death record data, we portrayed the risk environment by identifying neighborhoods most vulnerable to the opioid overdose epidemic in Lowell, and an expanding radius of risk over time. Rhodes defines the risk environment as “…the space – whether social or physical – in which a variety of factors interact to increase the chances of harm occurring” (Rhodes, 2009, Rhodes et al., 2005). Previous studies employed GIS to better understand the risk environment (Cooper et al., 2009), and identify and characterize hotspots related to the opioid crisis (Stopka et al., 2019, Stopka et al., 2012, Meyers et al., 2014). While an increasing number of studies have begun to analyze EMS data (Knowlton et al., 2013, Moore et al., 2017), few have capitalized on the power of local EMS data from a spatial analytical perspective (Pesarsick et al., 2019). Through descriptive maps, heat maps, and hotspot analyses, we identified neighborhoods where opioid-related outcomes clustered, helping to inform geographically targeted public health and harm reduction responses. Our findings demonstrate that systematic documentation, mapping, and spatial analysis of syringe discard data, apart from guiding clean-up efforts, provide a helpful proxy for spatial clustering of opioid overdoses, as well as other ORIs and injection-mediated risks (e.g., HIV and HCV transmission). These spatial analytical results can help to guide targeted harm reduction and disease screening responses, as well as linkage to treatment resources. Based on our results, the Downtown, Back Central, Lower Highlands, Acre, Centralville, and Lower Belvidere neighborhoods may be strong candidates for enhanced public health responses in Lowell. Such responses could include enhanced overdose education and naloxone distribution, which has been shown to decrease overdose risk in local communities (Walley et al., 2013), additional locations, days and hours of operation of SSPs, including weekend and evening hours, and bolstered street outreach to PWID in locations where syringe pick-up occurs. Davidson and colleagues, for instance, asked SSP participants “where were you and what time was it last time someone borrowed a needle from you?“ and ”where were you and what time was it last time you had to borrow a needle from someone else?“ They geocoded the responses and developed heat maps to identify locations in need of enhanced syringe services in Los Angeles, and inform conversations with local officials, funders, and policymakers (Davidson et al., 2011). Further, considering the overlapping spikes in opioid OD, HCV, and HIV among PWID in Lowell (Cranston et al., 2019, Alpren et al., 2020), it is important to consider the growing body of research focused on syringe-mediated syndemics (Bulled and Singer, 2011), and innovative analytical tools that aim to define “syndemic triangles” (Furr-Holden et al., 2016), or areas where drug treatment (and other prevention services) may be best positioned to reduce risk for overlapping and interacting injection-mediated health outcomes.

Our findings remind us that illicit drug crises are dynamic highly localized events (Holmberg, 1996, Friedman et al., 2004, Brady et al., 1992), not only in geographic space, with some local communities shouldering most of the burden, but also in time. Apart from the spatial clustering of syringe discard and opioid overdoses, we identified temporal trends that overlapped with seasonal patterns. These findings demonstrate the importance of targeting interventions to best align with the seasonal fluctuations in risk.

Place-based research is essential in understanding the risk environment in which drug injection occurs. The place-based approach assumes that geography matters in terms of its social, cultural, and institutional characteristics, which can inform policy interventions (Tempalski, 2007). Previous studies have demonstrated that the social, cultural, and political characteristics of different cities are likely to shape patterns of drug use, and the types of structural interventions that are adopted (Tempalski et al., 2007), impacting PWID health, and resulting in inequalities in risk environments to which PWID are exposed (Cooper et al., 2005). Place-based research, which facilitates identification of precise spatial extents of risk environments, and the development of tailored, local interventions that are mindful of local population characteristics, makes it uniquely qualified as an essential approach to assessing varying and geographically distinct opioid-related risks (Tempalski and McQuie, 2009).

Our findings should be considered in light of several limitations. First, EMS data do not capture all non-fatal overdoses and ORIs in Lowell, as EMS ORIs only include data from 9-1-1 calls. Patients who may have overdosed outside of the 9-1-1 system were not included. Still, these EMS data are the strongest available real-time data. Second, the location of documented fatal overdoses may be different from the location where the overdose actually occurred, particularly when a hospital is noted as the place of the recorded overdose. To address this limitation, we have incorporated injury and death locations from vital statistics data. Third, syringe discard reports are an indicator of a report being received by EMS, but a syringe was not always retrieved following each report. Further, the number of syringes retrieved at each site varies. Conversations with the EMS crews suggest 95% of the syringe discard reports resulted in at least 1 syringe being recovered. Fourth, we did not have access to hospital data for the current study, which could have facilitated a more precise understanding of fatal overdoses that occurred in the city, rather than in the hospital where they were ultimately reported. Our use of injury data, however, helped to address this issue. Finally, although we have clearly demarcated Lowell’s risk environments (Cooper et al., 2009, Tempalski and McQuie, 2009), we are limited in understanding the activity spaces (Martinez et al., 2014) of the city’s PWIDs. Specifically, we do not know how PWID in Lowell come to use their space and how they habitually move both within and outside the city. While such approaches were beyond the scope of this study, future research that assesses activity spaces (Martinez et al., 2014) may further elucidate Lowell’s risk environment (Rhodes, 2009).

We note several strengths of our study. The first is our innovative collaboration between municipal public health, local EMS, and academic partners, which facilitated a merger of front-line resources including data collection, policy planning, and spatial epidemiological research, to assess the risk environment, allowing us to look at the local opioid overdose epidemic through several lenses. We were also able to capitalize on the strengths of a local EMS data system that captured real-time data for ORIs and syringe discard. The temporal trends in ORI data and the spatial patterns in the syringe discard data mirrored trends and spatial patterns in opioid overdose data in Lowell, providing strong sentinel surveillance. These data, coupled with fatal overdose data, and GIS mapping and spatial analyses, allowed us to identify locations at highest risk of overdose and other drug-related comorbidities. Using EMS data in real-time has allowed Lowell to be nimble in its response to spikes in ORIs, allowing for quick responses and public alerts by public safety and public health officials to curb subsequent overdose risks (Garza and Dyer, 2016, Feathers, 2019). Application of these methods to additional real-time data, as well as development of dynamic forecasting models, are needed to help guide pre-emptive public health responses, much like predictive weather models guide emergency responses (Dodla et al., 2011).

As a result of our work and new harm reduction efforts in Lowell, in 2019 the Lowell Health Department developed and filled a City-funded position to respond to incoming pick-up requests for discarded syringes, to actively search public spaces for discarded syringes, and properly dispose of them. The staff member in this position works with community partners and the community at large to improve health and safety. Various datasets that our team oversees are shared publicly on a quarterly basis with the Mayor’s Opioid Task Force in Lowell to monitor progress with proper discard and inform harm reduction policy decisions.

## 6 Conclusion

Through an innovative collaboration between public health, EMS, and academic partners, we employed GIS, spatial and trend analyses of opioid-related outcome data enabling assessment of the risk landscape, identification of overlapping patterns, and provision of findings to inform local public health intervention efforts. Results produced through these analyses can inform targeted response efforts, guiding decision-makers to allocate the most needed public health and clinical resources to the neighborhoods that need them most.

## CRediT authorship contribution statement

**Thomas J. Stopka:** Conceptualization, Funding acquisition, Project administration, Supervision, Writing – original draft, Writing – review & editing. **Erin Jacque:** Methodology, Validation, Visualization, Writing – review & editing. **Jon Kelley:** Data curation, Methodology, Validation, Visualization., Writing – review & editing. **Lainnie Emond:** Data curation, Writing – review & editing. **Kerran Vigroux:** Validation, Writing – review & editing. **Wilson R. Palacios:** Conceptualization, Funding acquisition, Supervision, Validation, Writing – review & editing.

## Declaration of Competing Interest

The authors declare that they have no known competing financial interests or personal relationships that could have appeared to influence the work reported in this paper.

## References

[b0005] 2019 Massachusetts HIV/AIDS Epidemiologic Profile, Statewide Report (2020).

[b0010] Alpren, C., Dawson, E.L., John, B., et al., 2020. Opioid Use Fueling HIV Transmission in an Urban Setting: An Outbreak of HIV Infection Among People Who Inject Drugs-Massachusetts, 2015-201 Am. J. Public Health. 110(1):37-44. doi:10.2105/AJPH.2019.305366. 10.2105/AJPH.2019.305366 PMC6893347 31725317

[b0015] An Assessment of Fatal and Non-Fatal Opioid Overdoses in Massachusetts (2011–2015). http://www.mass.gov/eohhs/docs/dph/stop-addiction/legislative-report-chapter-55-aug-2017.pdf (2017).

[b0020] ArcGIS-Pro. How Kernel Density Works. 2020. Esri, Redlands, CA. Accessed February 14, 2020. https://pro.arcgis.com/en/pro-app/tool-reference/spatial-analyst/how-kernel-density-works.htm.

[b0025] ArcGIS-Pro. How Optimized Hot Spot Analysis Works. 2020. Esri, Redlands CA. Accessed February 14, 2020. https://pro.arcgis.com/en/pro-app/tool-reference/spatial-statistics/how-optimized-hot-spot-analysis-works.htm.

[b0030] Bearnot, B., Pearson, J.F., Rodriguez, J.A., 2018. Using Publicly Available Data to Understand the Opioid Overdose Epidemic: Geospatial Distribution of Discarded Needles in Boston, Massachusetts. Am J Public Health. 108(10):1355-1357. doi:10.2105/AJPH.2018.304583. 10.2105/AJPH.2018.304583 PMC6137768 30138067

[b0035] Brady, J.E., Friedman, S.R., Cooper, H.L., Flom, P.L., Tempalski, B., Gostnell, K., 2008. Estimating the prevalence of injection drug users in the U.S. and in large U.S. metropolitan areas from 1992 to 2002. J. Urban Health. 85(3):323-51. doi:10.1007/s11524-007-9248-5. 10.1007/s11524-007-9248-5 PMC2329751 18344002

[b0040] Buchanan D., Shaw S., Teng W., Hiser P., Singer M. (2003). Neighborhood differences in patterns of syringe access, use, and discard among injection drug users: implications for HIV outreach and prevention education. J Urban Health..

[b0045] Bulled N., Singer M. (2011). Syringe-mediated syndemics. AIDS Behav..

[b0050] Bureau UC. Explore US Census Data. September 2021, 2021. Accessed September 21, 2021, 2021. Available at: https://data.census.gov/cedsci/.

[b0055] Ciccarone D., Ondocsin J., Mars S.G. (2017). Heroin uncertainties: Exploring users' perceptions of fentanyl-adulterated and -substituted 'heroin'. Int. J. Drug Policy.

[b0060] Cooper H., Moore L., Gruskin S., Krieger N. (2005). The impact of a police drug crackdown on drug injectors' ability to practice harm reduction: a qualitative study. Soc. Sci. Med..

[b0065] Cooper H., Bossak B., Tempalski B., Des Jarlais D., Friedman S. (2009). Geographic approaches to quantifying the risk environment: drug-related law enforcement and access to syringe exchange programmes. Int. J. Drug Policy.

[b0070] Cranston K., Alpren C., John B. (2019). Notes from the Field: HIV Diagnoses Among Persons Who Inject Drugs – Northeastern Massachusetts, 2015–2018. MMWR Morb Mortal Wkly Rep..

[b0075] Davidson P.J., Scholar S., Howe M. (2011). A GIS-based methodology for improving needle exchange service delivery. Int. J. Drug Policy.

[b0080] Dodla, V.B., Desamsetti, S., Yerramilli, A., 2011. A comparison of HWRF, ARW and NMM models in Hurricane Katrina (2005) simulation. Int. J. Environ. Res. Public Health. 8(6):2447-69. doi:10.3390/ijerph8062447. 10.3390/ijerph8062447 PMC3138034 21776239

[b0085] Feathers, T., 2019. City issues warning following four fatal overdoses in 12 hours. Lowell Sun. March 22. Accessed July 29, 2019. Available at: http://www.lowellsun.com/breakingnews/ci_31753263/city-issues-warning-following-four-fatal-overdoses-12#ixzz5v4YtJjqP.

[b0090] Friedman S., Tempalski B., Cooper H. (2004). Estimating numbers of injecting drug users in metropolitan areas for structural analyses of community vulnerability and for assessing relative degrees of service provision for injecting drug users. J. Urban Health.

[b0095] Fryer, F., 2019. Outbreak of HIV fond among Boston Drug Users. Boston Globe. January 29, 2019. Accessed February 1, 2019. Available at: https://www.bostonglobe.com/metro/2019/01/29/boston-sees-outbreak-hiv-among-boston-drug-users/jtKq0FniDjRR1piAzgeCOJ/story.html.

[b0100] Furr-Holden D.M., Milam A.J., Nesoff E.D. (2016). Triangulating syndemic services and drug treatment policy: improving drug treatment portal locations in Baltimore City. Prog Community Health Partnersh..

[b0105] Furr-Holden C.D., Milam A.J., Nesoff E.D. (2016). Not in my back yard: A comparative analysis of crime around publicly funded drug treatment centers, liquor stores, convenience stores, and corner stores in one Mid-Atlantic City. J. Stud. Alcohol Drugs.

[b0110] Garza, A., Dyer S. Overdose Nation: A look at EMS' role in the U.S. opioid epidemic. JEMS. 11 2016;41(11):41-5. 29188960

[b0115] Holmberg S.D. (1996). The estimated prevalence and incidence of HIV in 96 large US metropolitan areas. Am. J. Public Health.

[b0120] Knowlton, A., Weir, B.W., Hazzard, F., et al., 2013. EMS runs for suspected opioid overdose: implications for surveillance and prevention. Prehosp. Emerg. Care;17(3):317-29. 10.3109/10903127.2013.792888. 10.3109/10903127.2013.792888 PMC3682796 23734988

[b0125] Martinez A.N., Lorvick J., Kral A.H. (2014). Activity spaces among injection drug users in San Francisco. Int. J. Drug Policy.

[b0130] MDPH, 2016. Number of Unintentional Opioid-Related Deaths by County, MA Residents: 2000-2015. Accessed December 20, 2016. Available at: http://www.mass.gov/eohhs/docs/dph/quality/drugcontrol/county-level-pmp/overdose-deaths-by-county-including-map-may-2016.pdf.

[b0135] MDPH, 2019. Number of Opioid-Related Overdose Deaths, All Intents by City/Town, 2014-2018. 2019. Accessed August 19, 2019. Available at: https://www.mass.gov/files/documents/2019/05/15/Opioid-related-Overdose-Deaths-by-City-Town-May-2019.pdf.

[b0140] MDPH, 2020. Hepatitis C Virus Infection 2014-2018 Surveillance Report. 2019. Accessed June 11, 2020. http://www.mass.gov/eohhs/gov/departments/dph/programs/id/.

[b0145] MDPH, 2021 Data Brief: Opioid-Related Overdose Deaths among Massachusetts Residents (2021). Accessed on June 8, 202Available at: https://www.mass.gov/doc/opioid-related-overdose-deaths-among-ma-residents-may-2021/download.

[b0150] Meyers D.J., Hood M.E., Stopka T.J. (2014). HIV and Hepatitis C mortality in Massachusetts, 2002–2011: spatial cluster and trend analysis of HIV and HCV using multiple cause of death. PLoS One.

[b0155] Milam A.J., Furr-Holden C.D., Harrell P.T., Whitaker D.E., Leaf P.J. (2012). Neighborhood disorder and juvenile drug arrests: a preliminary investigation using the NIfETy instrument. Am J Drug Alcohol Abuse..

[b0160] Moore P.Q., Weber J., Cina S., Aks S. (2017). Syndrome surveillance of fentanyl-laced heroin outbreaks: Utilization of EMS, Medical Examiner and Poison Center databases. Am. J. Emerg Med..

[b0165] Pesarsick J., Gwilliam M., Adeniran O., Rudisill T., Smith G., Hendricks B. (2019). Identifying high-risk areas for nonfatal opioid overdose: a spatial case-control study using EMS run data. Ann. Epidemiol..

[b0170] Rhodes T. (2009). Risk environments and drug harms: a social science for harm reduction approach. Int. J. Drug Policy.

[b0175] Rhodes T., Singer M., Bourgois P., Friedman S.R., Strathdee S.A. (2005). The social structural production of HIV risk among injecting drug users. Soc. Sci. Med..

[b0180] Rudd R.A., Seth P., David F., Scholl L. (2016). Increases in Drug and Opioid-Involved Overdose Deaths - United States, 2010–2015. MMWR Morb Mortal Wkly Rep..

[b0185] Sadler R.C., Furr-Holden D. (2019). The epidemiology of opioid overdose in Flint and Genesee County, Michigan: Implications for public health practice and intervention. Drug Alcohol Depend..

[b0190] Somerville N.J., O'Donnell J., Gladden R.M. (2017). Characteristics of Fentanyl Overdose - Massachusetts, 2014–2016. MMWR Morb. Mortal Wkly. Rep..

[b0195] Stopka T.J., Lutnick A., Wenger L.D., Deriemer K., Geraghty E.M., Kral A.H. (2012). Demographic, risk, and spatial factors associated with over-the-counter syringe purchase among injection drug users. Am. J. Epidemiol..

[b0200] Stopka T.J., Krawczyk C., Gradziel P., Geraghty E.M. (2014). Use of spatial epidemiology and hot spot analysis to target women eligible for prenatal women, infants, and children services. Am. J. Public Health.

[b0205] Stopka T.J., Amaravadi H., Kaplan A.R. (2019). Opioid overdose deaths and potentially inappropriate opioid prescribing practices (PIP): A spatial epidemiological study. Int. J. Drug Policy.

[b0210] Tempalski B. (2007). Placing the dynamics of syringe exchange programs in the United States. Health Place.

[b0215] Tempalski B., Flom P.L., Friedman S.R. (2007). Social and political factors predicting the presence of syringe exchange programs in 96 US metropolitan areas. Am. J. Public Health.

[b0220] Tempalski B., McQuie H. (2009). Drugscapes and the role of place and space in injection drug use-related HIV risk environments. Int. J. Drug Policy.

[b0225] Tookes H.E., Kral A.H., Wenger L.D. (2012). A comparison of syringe disposal practices among injection drug users in a city with versus a city without needle and syringe programs. Drug Alcohol Depend..

[b0230] United States Census Bureau. American FactFinder. Accessed: August 3, 2011. Available at: http://factfinder.census.gov.

[b0235] Walley A.Y., Xuan Z., Hackman H.H. (2013). Opioid overdose rates and implementation of overdose education and nasal naloxone distribution in Massachusetts: interrupted time series analysis. BMJ..

