# Supplementary material for: Examining the spatial risk environment tied to the opioid crisis through a unique public health, EMS, and academic research collaborative: Lowell, Massachusetts, 2008–2018
Source: Prev Med Rep. 2021 Oct 6;24:101591. doi: 10.1016/j.pmedr.2021.101591 (PMC8683861; doi:10.1016/j.pmedr.2021.101591)
Supplement: Supplementary data 1 [file mmc1.pdf]

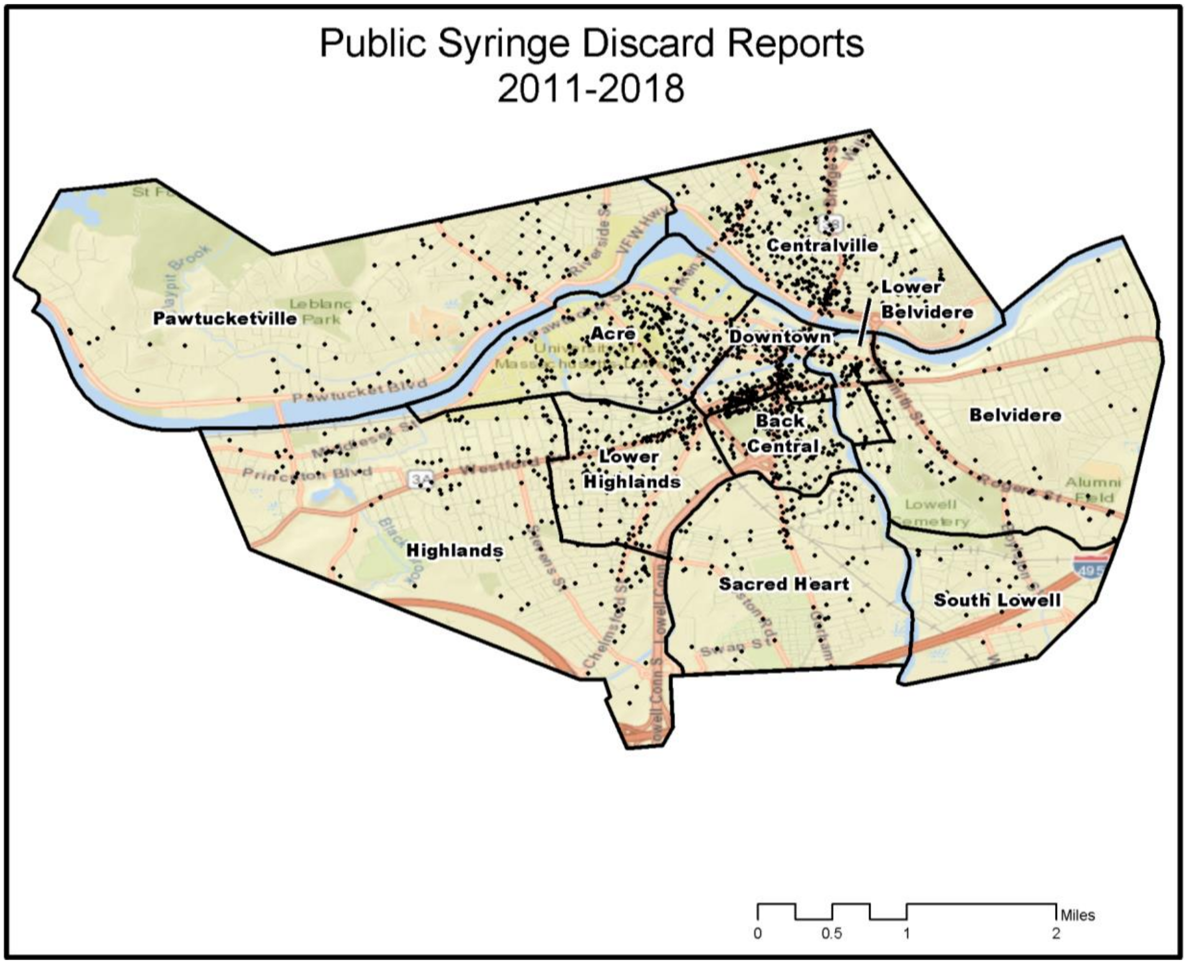

Public Syringe Discard Reports 2011-2019 were most concentrated in central Lowell, within the Downtown, Back Central, Lower Highlands, Acre, and Centralville neighborhoods. In some areas, the distribution of points appears to follow a linear distribution along transportation corridors/roads.

Appendix

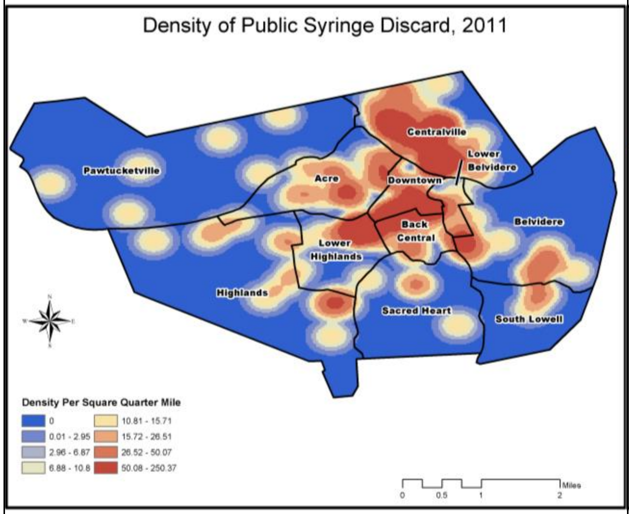
Density of Public Syringe Discard, 2011
Centralville
Lower Belvidere
Pawtucketville
Acre
Downtown
Back Central
Belvidere
Lower Highlands
Highlands
Sacred Heart
South Lowell
Density Per Square Quarter Mile
0
0.01 - 2.95
2.96 - 6.87
6.88 - 10.8
10.81 - 15.71
15.72 - 26.51
26.52 - 50.07
50.08 - 250.37
0 0.5 1 2 Miles

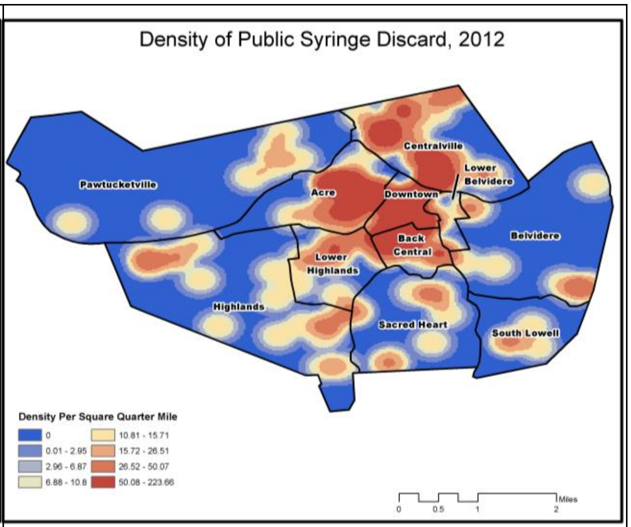
Density of Public Syringe Discard, 2012
Centralville
Lower Belvidere
Pawtucketville
Acre
Downtown
Back Central
Belvidere
Lower Highlands
Highlands
Sacred Heart
South Lowell
Density Per Square Quarter Mile
0
0.01 - 2.95
2.96 - 6.87
6.88 - 10.8
10.81 - 15.71
15.72 - 26.51
26.52 - 50.07
50.08 - 223.66
0 0.5 1 2 Miles

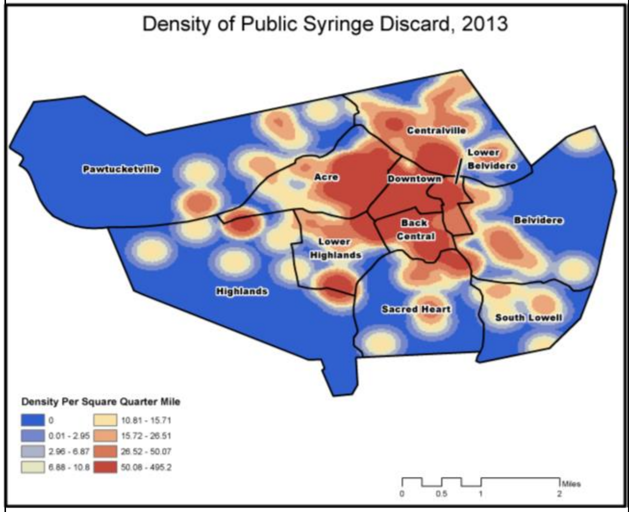
Density of Public Syringe Discard, 2013
Centralville
Lower Belvidere
Pawtucketville
Acre
Downtown
Back Central
Belvidere
Lower Highlands
Highlands
Sacred Heart
South Lowell
Density Per Square Quarter Mile
0
0.01 - 2.95
2.96 - 6.87
6.88 - 10.8
10.81 - 15.71
15.72 - 26.51
26.52 - 50.07
50.08 - 495.2
0 0.5 1 2 Miles

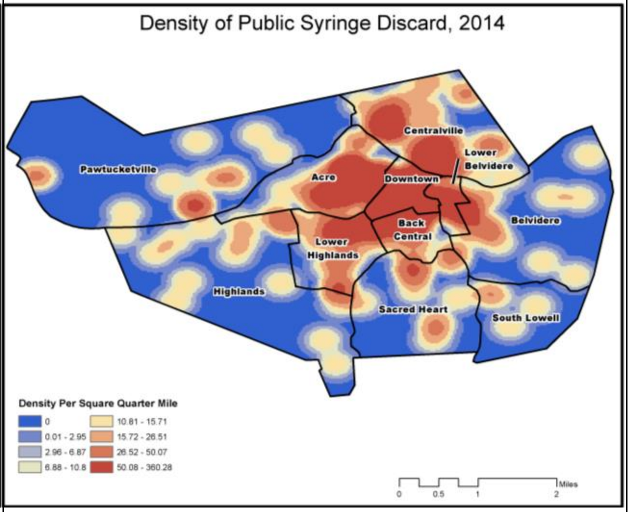
Density of Public Syringe Discard, 2014
Centralville
Lower Belvidere
Pawtucketville
Acre
Downtown
Back Central
Belvidere
Lower Highlands
Highlands
Sacred Heart
South Lowell
Density Per Square Quarter Mile
0
0.01 - 2.95
2.96 - 6.87
6.88 - 10.8
10.81 - 15.71
15.72 - 26.51
26.52 - 50.07
50.08 - 360.28
0 0.5 1 2 Miles

Appendix

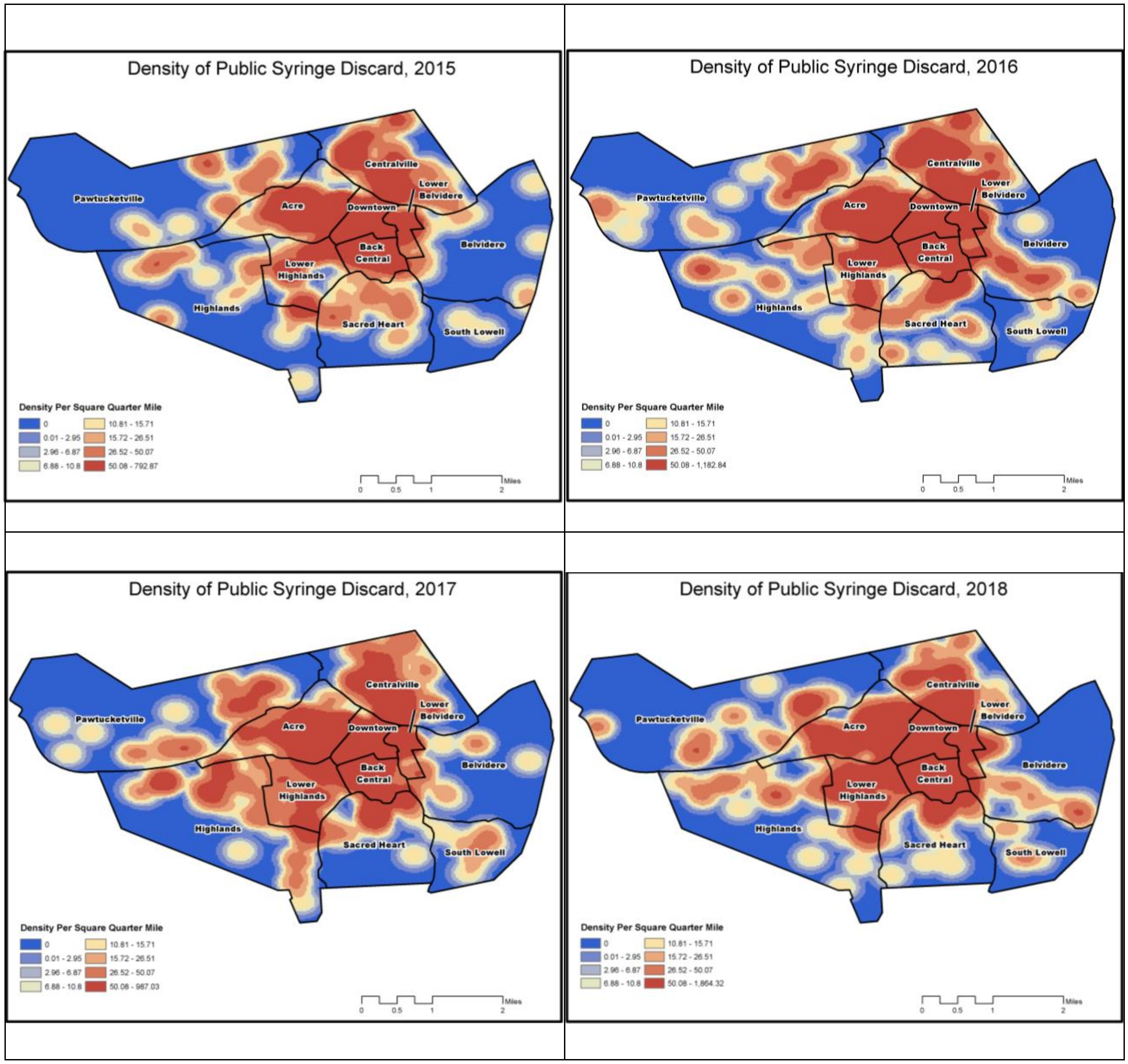

Public Syringe Discard Reports 2011-2019: We calculated Kernel density estimates analysis for each year from 2011-2018.

Appendix

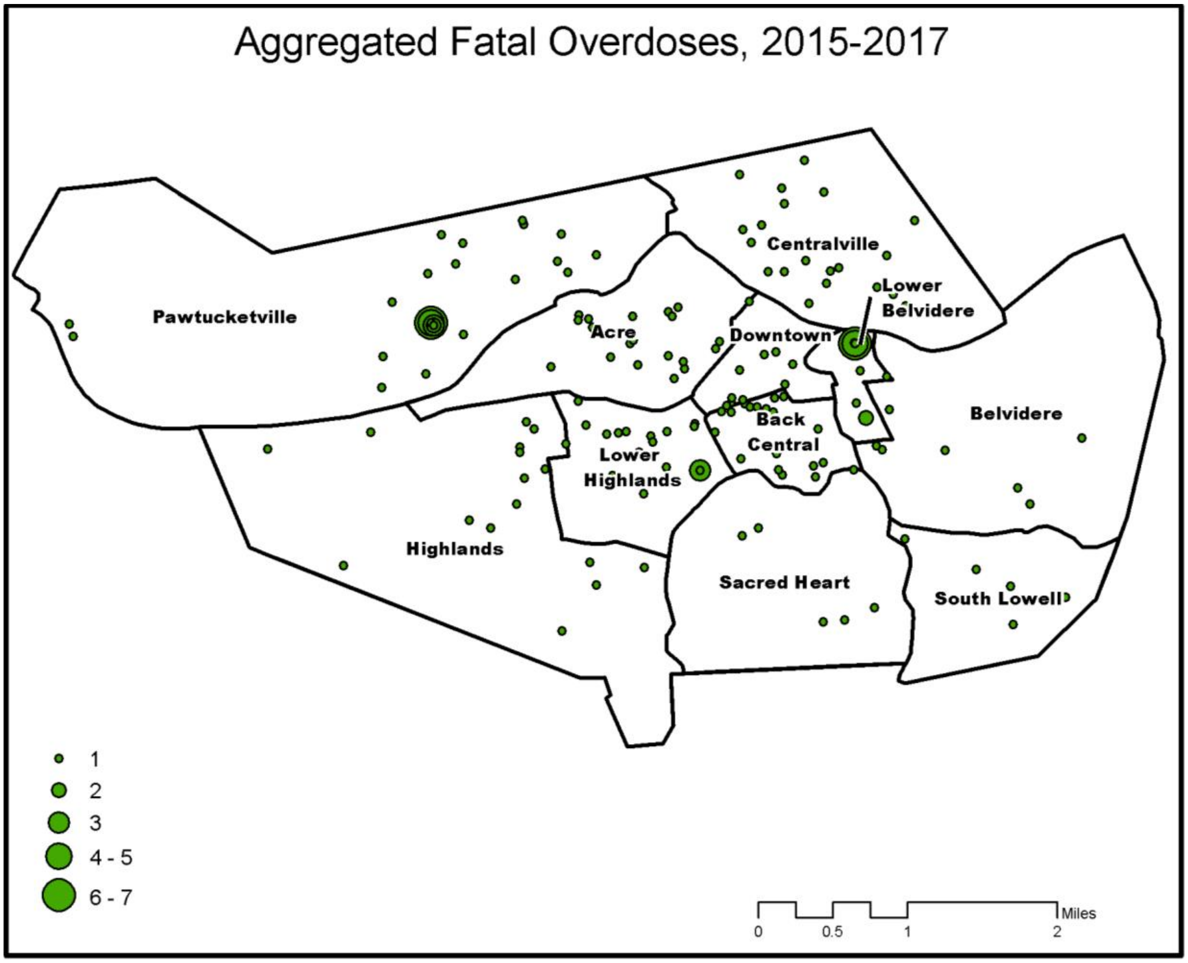

Fatal Overdose 2015-2017: Some fatal overdose locations shared the same street address. To more accurately view the number of fatal overdoses, we used graduated symbols to highlight the locations where multiple fatal overdoses were reported.

Appendix

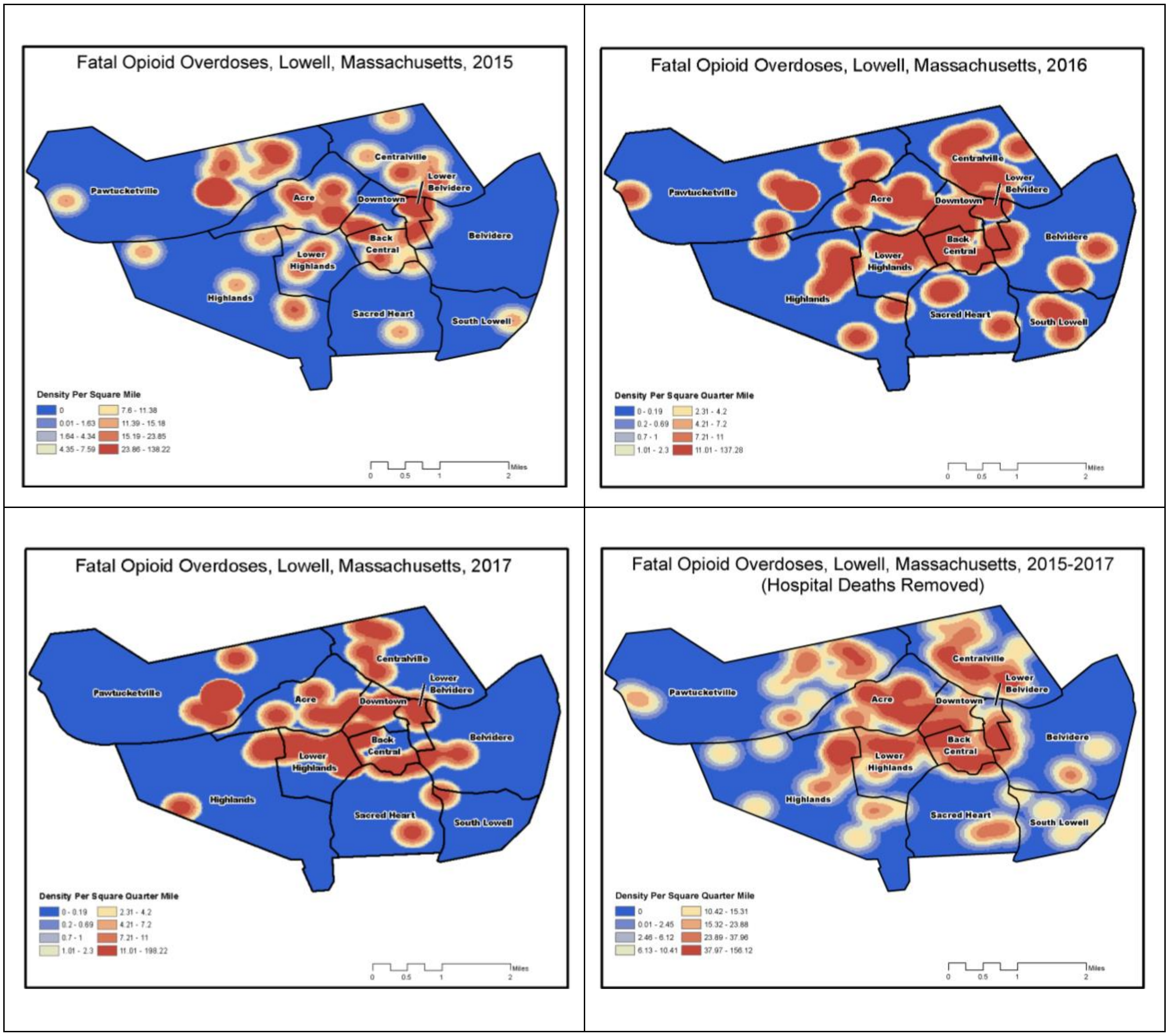

We created heat maps for 2015-2017 for all fatal overdose death locations and fatal overdose death locations with hospital points removed.

Appendix

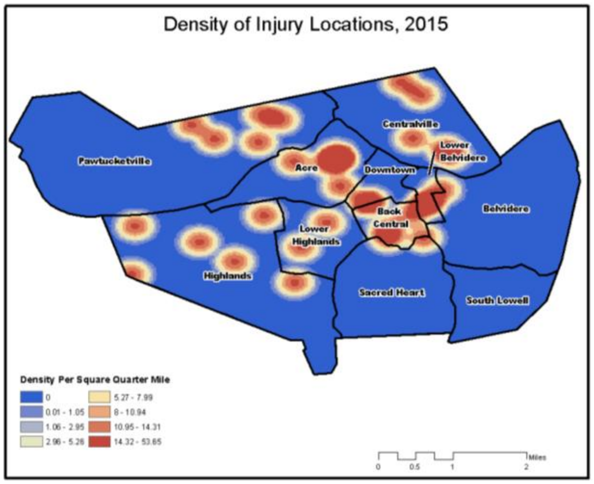

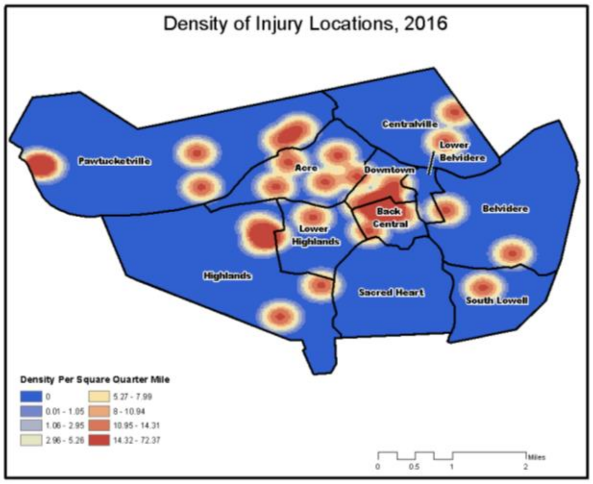

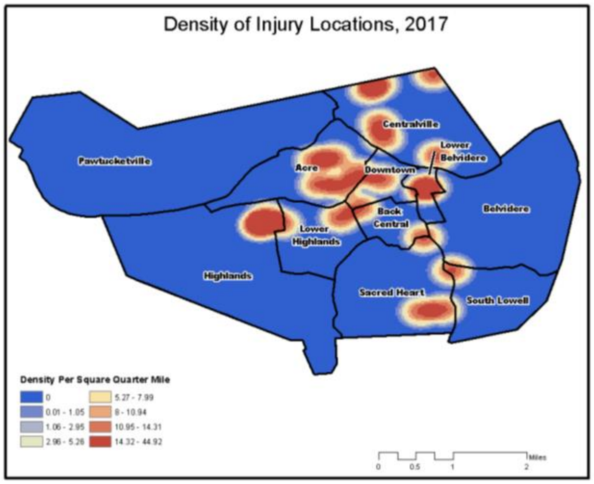

We calculated Kernel density estimates for 2015-2017 for decedents injury location for use in the multi-variable composite density map.
